# BIOLOGICAL AGE REDUCTION AND LIFESPAN EXTENSION UPON YOUTHFUL CIRCULATION EXPOSURE

**DOI:** 10.1093/geroni/igad104.1942

**Published:** 2023-12-21

**Authors:** Bohan Zhang

**Affiliations:** Brigham and Women’s Hospital, Boston, Massachusetts, United States

## Abstract

We performed extended (3-month) Heterochronic Parabiosis (HPB), followed by a 2-month detachment period of anastomosed pairs. Old detached mice exhibited improved physiological parameters and lived longer than control isochronic mice. HPB drastically reduced the biological age of blood and liver based on epigenetic analyses across several clock models using multiple independent platforms; remarkably, this rejuvenation effect persisted even after 2 months of detachment. Transcriptomic and epigenomic profiles of anastomosed mice showed an intermediate phenotype between old and young, suggesting a global multi-omic rejuvenation effect. In addition, old HPB mice showed gene expression changes opposite to aging, but akin to several lifespan-extending interventions. Altogether, we reveal that long-term Heterochronic Parabiosis can decrease the biological age of mice, in part through long-lasting epigenetic and transcriptome remodeling, culminating in the extension of lifespan and healthspan.

